# Recommendations for the Development of Psychological Smartphone Applications in the Context of Bariatric Surgery: Focus Groups with Professionals and Patients

**DOI:** 10.1007/s10880-024-10039-z

**Published:** 2024-08-20

**Authors:** Verónica Martínez-Borba, Alba Quilez-Orden, Vanessa Ferreres-Galán, Christian López-Cruz, Jorge Osma, Laura Andreu-Pejó

**Affiliations:** 1 Instituto de Investigación Sanitaria de Aragón, Avenida San Juan Bosco, 13, 50009 Zaragoza, Spain; 2 Unidad de Salud Mental Moncayo, Calle Cortes de Aragón, 14, 50500 Tarazona, Spain; 3 Unidad de Salud Mental del Hospital Comarcal de Vinaròs, Avenida Gil De Atrosillo S/N, 12500 Vinaròs, Spain; 4 Universitat Jaume I, Avenida Vicente Sos Baynat S/N, 12071 Castellón, Spain; 5 Universidad de Zaragoza, Calle Atarazanas, 4, 44003 Teruel, Spain

**Keywords:** Bariatric surgery, Emotional regulation, Psychological intervention, Mobile applications, Focus group

## Abstract

To explore the experiences and preferences of patients and healthcare professionals regarding the development of an app to provide psychological intervention to improve emotion regulation in the context of bariatric surgery (BS). Sixteen people (6 patients who underwent BS and 10 professionals) participated in two separate focus group sessions. We performed a content analysis of transcribed focus group discussions to extract and organize categories, subcategories and areas. Both sets of stakeholders provided information about how to develop and implement an app. According to participants’ comment, content should include information (i.e., nutrition, exercise) and emotional regulation skills. Patients and professionals mentioned that the app should include visual information, continuous emotional assessments and peer contact. It was also mentioned that the app should be used before and after BS and its contents should be developed by a multidisciplinary team (i.e., collaboration of endocrinologist, nutritionists and psychologists). Participants in both focus groups considered technology to be useful in the context of BS, especially as part of blended interventions (combining face-to-face and online sessions). Patients and professionals seem to be receptive towards the use of technology in a BS context. Specific recommendations are identified for designing and implementing app solutions for BS. More efforts should be made in the future to develop and implement evidence-based apps according to patients and professionals’ needs.

## Introduction

Obesity is a serious public health problem worldwide due to its high prevalence and its health, social, and economic impact (Spirou et al., 2020). According to the World Health Organization, obesity rates have tripled during the three last decades, and 43% of adults worldwide were overweight in 2022 (World Health Organization, 2024). In Spain, it is expected that by 2030 up to 80% of men and 55% of women will have obesity or overweight (Hernáez et al., 2019). Individuals with obesity are at increased risk for physical difficulties and emotional comorbidities, that may, in turn, affect their global functioning. For instance, Simon et al., (2006) estimated that people with obesity have a 25% increased risk of anxiety and mood disorders (also known as emotional disorders; Bullis et al., 2019). Different explanatory mechanisms for the bidirectional relationship between obesity and emotional problems have been considered. For example, people with emotional disorders may suffer from symptoms such as increased appetite, decreased behavioral activation, or use of medication that facilitates weight gain. In the opposite direction, people with obesity may be adversely affected by weight stigma or by the difficulty of participating in rewarding or pleasurable activities, which may also increase emotional symptoms (Simon et al., 2006).

A range of behavioral, nutritional, medical, and surgical interventions are available to individuals who are seeking professional support for weight management (Bean et al., 2008). Bariatric surgery (BS), including open or laparoscopic roux-en-Y gastric bypass, sleeve gastrectomy, and adjustable gastric banding (Jumbe et al., 2017), is recommended for individuals with clinically severe obesity (Sarwer & Heinberg, 2020). After BS, most patients lose a significant amount of weight in the short term; however, between 20 and 50% of patients begin to regain weight after the first year and a half to two years (Sarwer & Heinberg, 2020).

Weight regain after BS is associated with physiological and behavioral factors (e.g., poor stress management or problem-solving skills), in the absence of ongoing instruction to facilitate incorporation of learned changes into long-term routines (Sarwer & Heinberg, 2020). In addition, the evidence indicates that emotional factors, such as anxiety, depression, and emotional eating, are among the most important contributors to weight regain after BS (Hjelmesæth et al., 2019; Lai et al., 2021). Psychological interventions have been developed to improve the psychological well-being of patients who undergo BS, in part to help maintain weight loss following surgery. Cognitive behavioral skills and strategies such as goal setting, psychoeducation, self-monitoring, stimulus control, cognitive restructuring, problem solving, and using reinforcement and relapse prevention could improve the frequency of uncontrolled eating, quality of life, anxiety, depression and social functioning (Lauren et al., 2020).

The Unified Protocol for the Transdiagnostic Treatment of Emotional Disorders (UP) is a cognitive behavioral psychological intervention that addresses shared etiological and maintenance factors for emotional disorders by providing training on adaptive emotion regulation skills (Barlow et al., 2018). One advantage of this intervention is its applicability across a wide variety of presenting concerns and patient populations (Osma et al., 2021). Recently, our team adapted and implemented a UP-based group psychological intervention for patients who had undergone BS in the Spanish National Health System (Ferreres-Galán et al., 2022). Results from this study suggested that the UP is a feasible intervention to improve emotional regulation in these patients. However, some of the limitations of the Spanish National Health System are the limited number of professionals and the long waiting lists to receive mental health care (Osma et al., 2022a, 2022b), which may compromise the dissemination of evidence-based psychological interventions. As a solution, recent investigations have focused on developing and implementing cost-effective interventions such as electronic health (eHealth) and mobile health (mHealth) applications, or apps, to provide psychological interventions with diverse intensity formats (i.e., from self-applied programs to blended interventions combining face-to-face and online sessions). Systematic reviews have found that eHealth/mHealth programs could be a cost-effective solution to provide physical and psychological assessments and interventions in populations with physical and/or emotional conditions (Iribarren et al., 2017; Kählke et al., 2022; Mitchell et al., 2021). eHealth and mHealth apps can also complement to face-to-face therapy, increasing its cost-effectiveness (Kumar et al., 2018).

mHealth has been used with promising results in the management of emotional disorders and comorbid health conditions (Barbosa et al., 2021), and also in the improvement of different outcomes in patients undergoing BS (Messiah et al., 2020). Apps can be used to deliver physical activity interventions, psychological education, and cognitive behavioral interventions to improve positive behavior changes, weight loss, promote exercise adoption, and reduce visits to emergency health services (Heuser et al., 2021; Messiah et al., 2020). However, a fundamental issue in the development of mHealth is to include the final users in its design, which is known as user-centered design. Specifically, this approach implies taking into account user comments on the desired features in the app in order to improve its usability and acceptability, which may improve user adherence and satisfaction (Molina-Recio et al., 2020). Recent studies suggest that it would also be interesting to consider the comments of the professionals involved in the interventions (Alqahtani & Orji, 2020) because it may increase the trustworthiness of the tool, its effectiveness, and its alignment with the needs of stakeholders (Alqahtani & Orji, 2020; Dekker & Williams, 2017).

The aim of this study was to explore the needs, experiences, and preferences of Spanish patients and professionals regarding the development of an app to provide UP-based psychological intervention to improve emotional regulation skills in patients who are waiting to receive BS or have already undergone BS. We hope that results derived from this study will help to develop a future app solution to provide psychological care tor BS patients.

## Methods

### Participants

We contacted 18 people (7 patients and 11 professionals) to participate in the focus group sessions. In the end, two participants (one patient and one professional) were not able to attend the sessions due to scheduling conflicts. Consequently, a total of 16 participants were enrolled in this study. Two different subsamples were selected according to sample convenience. First, we aimed to recruit patients (*n* = 6) who had already participated in a face-to-face UP intervention to learn emotional regulation skills after BS (Ferreres-Galán et al., 2022) because they already know the UP and can provide recommendations about how to translate the intervention to an online format. Participants were recruited from a Spanish public hospital called Hospital Comarcal de Vinaròs. Participants were referred to the mental health unit from the endocrinology unit for presenting difficulties or symptomatology related to emotional disorders. As described in the study (Ferreres-Galán et al., 2022) participants in the UP psychological program had to meet criteria for at least one emotional disorder, be waiting for BS or post-BS, understand Spanish or Catalan, and be available to attend face-to-face group sessions. After participating in the UP program, the psychologist contacted them again to offer them the possibility to take part in the focus group session. The BS received by all focus group participants was gastric bypass, and the median number of months from surgery to completion of the group was 46 months, so that all participants received the BS before starting the UP-based psychological program.

Second, we also aimed to recruit professionals (*n* = 10) from different specialties (i.e., psychologists, physicians, etc.) who were selected because they worked with BS patients (i.e., endocrine, nutritionist), or because they had clinical experience applying the UP in people with emotional disorders with or without additional health conditions. The professionals were not experts in the development of apps, so they were not asked about technical aspects of apps but rather how they think that the UP can be provided through an app and exercises that should be incorporated to help BS patients to improve their health behaviors.

### Procedure

This study was conducted in a Public Hospital in Spain. Approval was obtained from the Research Ethics Committee of the Hospital Comarcal de Vinaròs. This study was performed in line with the principles of the Declaration of Helsinki.

Focus groups were selected as the most convenient methodology to obtain participants’ feelings and opinion with regard to a specific topic (Lavraskas, 2008). Separate focus groups for patients and professionals were conducted to allow both subsamples to freely express themselves. Participants in both focus groups were contacted by telephone by one member of the research team who explained the main objective of the study and encouraged participants to enroll in this qualitative research. Participants interested in collaborating received written information by mail and signed informed consent allowing them to participate in the online focus group and the recording of the session. For ethical purposes, recordings were deleted once they were transcribed.

Both focus groups lasted around 2 h each and were mediated by an experienced researcher (J.O*.*) who had not previously met the participants. Only one observer was present in the session. An online platform (Cisco Webex) was used to conduct the online focus group through video calls. At the beginning of the session, the mediator explained the aim of the study and the development of the session and addressed any participants questions or concerns about the study. At the end of the focus group, the researcher checked the recordings, and no additional notes were taken. As no problems were detected, the interviews were not repeated.

### Measures

Two similar semi-structured interview guides were developed to cover specific research questions of interest for the research team. The mediator of the focus groups elaborated the questions taking into account the general research aim of the study and the specific research questions (Bryman, 2012). The first draft of the interview guide was revised and approved by all the research members (Appendix 1). As can be seen in Appendix 1, both interviews cover the same topics: use of technologies for mental health purposes, opinion about mobile applications for health purposes, use of mobile applications for physical health, elements to include in mHealth, and barriers and facilitators for the use of mHealth.

### Data Analysis

The consolidated criteria for reporting qualitative research (COREQ; Tong et al., 2007) was followed to describe the results derived from this study (Appendix 2).

We used the Social Package for Social Sciences (SPSS; IBM Corp, 2013) to explore descriptive characteristics of the sample. Then the MAXQDA software (Kuckartz & Rädiker, 2019) was employed to conduct the content analysis of the focus groups. First, the recordings were transcribed word by word. The procedures described by Schreier (2012) to conduct qualitative content analysis of our focus groups were followed to analyze the focus groups’ information. This approach facilitated the construction of a hierarchical coding schedule that allowed the extraction of general categories, which were composed of more specific subcategories and their corresponding areas.

To favor inter-rater reliability, two independent researchers extracted the categories, subcategories and areas. In case of disagreement, a third researcher was consulted (triangulation process). Finally, we calculated the Cohen’s kappa between the first draft of the data extraction and the final version to analyze the reliability of our qualitative analyses. Participants in the focus groups did not provide feedback on the findings.

## Results

### Participant Characteristics

As shown in Table 1, patients involved in this study were women around 53 years old who underwent BS an average of 46 months ago. Most of them achieved high school education, were in a stable romantic relationship, and were currently employed. Participating professionals included endocrinologists, psychologists, psychiatrists, and a nutritionist, with a mean of 18 years of expertise in the context of BS.

**Table 1:** Sociodemographic characteristics of participants (*n* = 16)

Variable	Patients (*n* = 6)	Professionals (*n* = 10)
	Mean (*SD*)	Mean (*SD*)
Age	53.00 (8.39)	45.90 (10.07)
Months since surgery/Years of expertise	46.00 (10.34)	17.90 (8.65)
	Proportion (%)	Proportion (%)
Educational level		
Elementary	16.67	0
High School	83.33	0
University studies	0	100
With a stable partner	66.67	70
Currently working	66.67	100
Professional specialty		
Endocrine	–	20
Psychologist	–	30
Psychiatrist	–	40
Nutritionist	–	10

### Data Extraction Reliability

As observed in Table 2, agreement ranged from 75 to 88%, which indicates a moderate agreement between the first extraction of the data and the final version of the extraction.

**Table 2:** Data extraction reliability

FG session	Areas		Subcategories		Categories	
	%	Cohen’s kappa	%	Cohen’s kappa	%	Cohen’s kappa
Patients	85.07	0.42, moderate	88.23	0.43, moderate	80.00	0.54, moderate
Professionals	85.00	0.41, moderate	83.33	0.55, moderate	75.00	0.50, moderate

*FG* focus group, *%* Percentage of agreement between the draft version and the final version of data extraction

### Categories, Subcategories, and Areas Extracted

As shown in Tables 3 and 4, content analysis resulted in the extraction of 32 areas, 10 subcategories, and 3 categories from the professionals’ focus group, and 59 areas, 16 subcategories, and 4 categories from the patients’ focus group (for a detailed description, see Appendices 3 and 4).

**Table 3:** Extraction of areas, subcategories and categories from the patients’ focus groups

Categories (*n* = 4)	Subcategories (*n* = 16)	Areas (*n* = 59)
App design	Content	Useful information Emotional regulation Crisis intervention Nutrition
	Functionality	Logs Audio-visual (video vs. text) Reminders Reinforcement Chat-social support Notes Questionnaires Guided Personalization Weight graph Calendar Shopping cart
	Duration of monitoring	Before surgery After surgery Continuous use Frequency of app use
	Professionals’ involvement	Surgeon Endocrinologist Psychiatrist and psychologist Dietician/nutritionist Traumatologist
App implementation	Barriers	App not interesting to the user
	Solutions	Audio Videos Gamification
	Population profile	General population
Opinion on technology	Usefulness	Use for psychological treatment
	Face-to-face benefits	Commitment to therapist Personalization Motivation/Reinforcement Credibility Mobility
	Face-to-face disadvantages	Shame Displacement
	App advantages	Immediacy Perseverance Personalization Distance learning
	App disadvantages	Veracity of data Lack of personalization No mobility
	Format preferences	Blended
Use of technology	Type of device	Mobile Tablet Television and computer
	Period of use	1 h 3–4 h 4–5 h 10 h
	Target	Information Leisure time/multimedia Employment Social networking Sport and food Mental Health

**Table 4:** Extraction of areas, subcategories and categories from the professionals’ focus groups

Categories (*n* = 3)	Subcategories (*n* = 10)	Areas (*n* = 32)
App design	Content	Psychoeducation Emotional regulation Medical aspects Useful information Exercise
	Functionality	Logs Questionnaires Weight graph Audiovisual Chat Calendar
	Duration	Time of intervention Duration of monitoring
App implementation	Barriers	Overload Motivation Lack of human contact
	Solutions	Initial professionals’ contact Explain benefits Testimonials Notifications Gamification-Reinforcement Personalization Professionals
	Intention to use	Recommendation
Opinion on technology	Usefulness	Apps use
	Face-to-face disadvantages	Bias Time
	Apps advantages	Accessibility Self-monitoring Information
	Format preference	Face-to-face Blended

#### App design

Four subcategories were mentioned in relation with app design:

##### Content

Participants mentioned some important content that should be included in the app, namely information and emotional regulation:

##### Information

Patients recommended inclusion of relevant information in the app, such as procedures and consequences of BS. Professionals specified that some nutritional information (i.e., how to prepare meals or a shopping list by a dietitian) should be provided.

##### Emotional Regulation

Both sets of stakeholders indicated that the app should include training in emotional regulation skills. Professionals specified that is important to provide patients with psychological skills to cope with emotional eating or food transgression. Participants postulated the need to include skills to help patients to manage unpleasant emotions such as sadness or boredom.

Additionally, only professionals mentioned the need to include information on psychoeducation, exercise, and medical aspects.

#### Functionality

Six areas were mentioned related to the app’s functionality:

##### Logs

In both focus groups, it was mentioned that it would be interesting to include logs in the app. A professional stated, “*I think that the app should include daily records of how these people feel on a day-to-day basis.*”

##### Audio-visual

Participants highly recommended using videos to provide information.

##### Chat Support

Professionals and patients considered relevant that apps offer the possibility to be in contact with other patients.

##### Questionnaires

During the two focus groups, it was recommended that the app includes the assessment of emotional components.

##### Calendar

Similar to logs, a calendar was also a functionality suggested by professionals and patients. To name an example, a professional recommended, “*With a calendar, a patient can remember the time elapsed since surgery.*”

##### Weight Graph

This area was mentioned by patients and professionals as a useful tool to visually monitor weight progress.

Apart from these areas, patients also discussed ideas as including reinforcements (i.e., congratulations or rewards for achievements), allowing the patient the opportunity to individualize components of the app, and reminders to facilitate the participant to engage with the app.

#### Duration

Timing of use of the app was mentioned by both sets of stakeholders. Some patients and professionals mentioned the need to use the app “before” or “after” BS while other patients expressed the need to use the device “constantly.” Professionals even highlighted the need to use apps in the long term: “*Patients usually gain weight two years after the BS (…) we should address it.*”

#### Professionals’ Involvement

The need to include a multidisciplinary team (i.e., endocrinologist, psychologist, etc.) was mentioned only by patients.

#### App Implementation

Four subcategories were mentioned with regard to app implementation:

##### Barriers

Participants expressed their concerns about the implementation of the UP through an app. Patients talked about app being not interesting to the user as an area that may hinder the implementation of the app. Professionals discussed the “overload,” “motivation,” and “lack of human contact”.

##### Solutions

Some solutions to overcome the low adherence with the app were proposed. Providing participants with gamification was a solution mentioned in both focus group, while offering videos and audios was suggested only by patients. Professionals stated that offering personalization and avatar customization, initial professional contact, an explanation of the benefits, testimonials, and notifications may help to engage users.

##### Population

Only the patients indicated that the app could be useful not only for BS patients but also for the general population.

##### Intention to Use

Only professionals gave their opinion about their intentions to use the app. It seemed that professionals would recommend the use of the app to their patients if they could first check that it works well and is useful for them.

#### Opinion on Technology

##### Usefulness

Professionals indicated that they know and use some apps that could be useful for their patients. In general terms, patients stated that apps could be useful if they are used properly.

##### Face-to-Face Benefits

Patients mentioned some arguments in favor of face-to-face interventions, namely the increased commitment to the therapist, the possibility to offer personalization, increased patient motivation (i.e., feeling more motivated after seeing the therapists face-to-face because with her/his feedback patients are encourage to maintain their progress), higher reliability (i.e., face-to-face programs to have more credibility than online solutions), and the promotion of mobility.

##### Face-to-Face Disadvantages

Patients highlighted that shame and travel are barriers for face-to-face treatments. In turn, professionals mentioned bias and use of time as limitations of traditional face-to-face models of care.

##### App Advantages

Perceived benefits of using mHealth included immediacy, perseverance, personalization, and facilitating distance learning/working from home for patients with mobility problems (patients’ focus group) and accessibility, self-management, and information (professionals’ focus group).

##### App Disadvantages

Some patients expressed their concerns with regard to the use of mHealth including data veracity, lack of personalization, and lack of mobility.

##### Format Preferences

Patients and some professionals agreed that a blended delivery format would be the most useful. On the other hand, other professionals noted that an app could not replace contact with a professional.

#### Use of technology

Patients (not professionals) stated that the most widely used devices were smartphones, tables, television, and the computer. In relation with duration of use, patients reported using technological devices daily for 3–4 h. The most common reasons for using technology were to search for information, leisure time, work activities, social networks, exercise, nutrition and mental health.

## Discussion

This study aimed to explore the opinions and preferences of patients and professionals regarding the development of an app to provide a psychological intervention to BS patients. We found that professionals and patients considered it extremely important to address psychological issues and emotional regulation in BS patients. Participants agreed on the basic information and functionality that should be covered by the app which may favor the collaborative use of these apps. We will briefly discuss some of the most important recommendations provided during the focus group sessions.

Previous studies, as well as ours, highlighted the relevance of training emotion regulation in BS patients because it has been associated with weight maintenance after BS (Hjelmesæth et al., 2019). Additionally, participants suggested that the app should provide information about nutrition, exercise, and medical procedures (e.g., Breuing et al., 2022).

In relation with timing of use of the app, participants felt that app use could be beneficial both before and after BS. Using an app before BS may help users to improve nutritional knowledge and other BS outcomes such as eating behaviors (Paul et al., 2021; Sherf-Dagan et al., 2018). We believe that the app proposed in this work could serve to provide information about how to prepare for BS, promote adherence to surgical guidelines, develop a weight loss plan, (i.e., workout routine, diet plans, and recipes according to their needs), and develop emotion regulation skills to improve physical and psychological outcomes after the surgery.

Professionals recommended that follow-up with the app should be extended long-term. Previous studies have suggested that lifelong follow-up of BS patients can be beneficial by increasing the professionals understanding of the progression of this population and by superior long-term outcomes in these patients (DeMeireles et al., 2019). For this reason, the app may serve to conduct regular physical and psychological assessments which help the patients and professionals to follow the progresses and difficulties. However, it is also important to note that previous studies have found high dropout rates when longitudinal follow-up of BS patients is conducted (The LABS Consortium Retention Writing Group et al., 2013). Reasons for discontinuing include lack of time, work responsibilities, or long distances to the hospital (The LABS Consortium Retention Writing Group et al., 2013). Apps have the potential to offer alternative assessments and intervention programs reducing these issues (DeMeireles et al., 2019). We think that the inclusion of technology to conduct long-term interventions could be especially useful after BS. Furthermore, patients in our focus group valued a multidisciplinary approach. Previous qualitative studies have similarly found that BS patients appreciate having access to different professionals, including a dietitian, physiotherapist, and psychologist (Tolvanen et al., 2021). Consequently, we propose the app to be developed and supported by a multidisciplinary team. This way, different professionals could help patients access relevant, evidence-based information and resources in the app.

With regard to the functionality that should be used to provide this content, professionals and patients recommended paying special attention to interactive assessment features such as ecological momentary assessments through logs, questionnaires, and a weight graph. Furthermore, and similar to previous findings (Conceição et al., 2020), professionals and patients in our focus group asked for chatrooms and forums to facilitate social support. Recommendation for peer contact will inform our further development of an app for BS patients.

An additional category mentioned in both focus groups sessions was app implementation. According to our participants’ reports, apps that are not sufficiently interesting or stimulating to the user, lack of human contact, and make excessive demands could result in a low adherence with the app. Similar to recent investigations (Osma et al., 2022a), our participants suggested that these issues could be reduced if the app were to allow contact with professionals, be customizable, offer gamification, and provide visual and dynamic information.

The last category shared by professionals and patients was advantages and disadvantages of online and face-to-face formats. As in previous studies (Andersson & Titov, 2014; Wentzel et al., 2016), patients noted both benefits of face-to-face interventions (e.g., commitment) and inconveniences of face-to-face interventions (e.g., traveling to the hospital). On the other hand, and in congruence with literature (Andersson & Titov, 2014), advantages of app solutions were also mentioned (e.g., having immediate responses). According to this information, it seems that both formats are equally useful and problematic. Combined interventions or blended format could be a solution (Webb & Orwig, 2015), integrating the advantages of the online and face-to-face formats and making the app a suitable tool for the therapist and the patient (Wentzel et al., 2016). The app proposed in this work could serve to provide a complete psychological intervention based on the UP. Depending on the characteristics of the patients and the health service resources, they could use the app in a self-administered way or as a complement to face-to-face sessions with the therapist (i.e., reinforce the techniques or practice the exercises).

Results from this study should be also understood in the context of some limitations. First, although focus groups are the mainstream for obtaining experiences and feelings from participants that shared a specific context (Lavraskas, 2008), it is also worth pointing out that interactions during focus groups may lead to socially expected responses (Bryman, 2012). Additionally, a small sample size was obtained in this study and data saturation was not possible. Although the professionals’ focus group was multidisciplinary, it included a limited number of providers from each discipline (e.g., participation of only one nutritionist). For this reason, conclusions derived from this study should be interpreted with caution. Finally, despite the potential use of the UP as a psychological transdiagnostic intervention for people undergoing BS (Ferreres-Galán et al., 2022), there is still a need for rigorous studies to determine the utility of this intervention in this population.

While acknowledging the aforementioned limitations, to the best of our knowledge, this is the first study that explores the opinions and recommendations of patients and professionals with regard to the development of an app to provide psychological care in the context of BS. One of the strengths of this study is the inclusion of both sets of stakeholders, patients and professionals. Additionally, it has been observed that psychological research on BS patients tends to rely mainly on quantitative studies that do not allow insights into the experience of BS from patients perspectives (Jumbe et al., 2017). As a solution, we included a necessary qualitative approach that allowed the analysis of the direct experiences of both sets of stakeholders.

Some promising results have been found in the management of emotional disorders in post-BS patients (Ferreres-Galán et al., 2022). However, the limitations of current face-to-face psychological interventions make it difficult to access evidence-based psychological interventions. mHealth has been proposed as a solution to overcome some of the barriers of onsite interventions, but the necessity to include final users’ needs in the development of such solutions (Garrido et al., 2019) has also been stated. Future efforts should be conducted to develop and implement evidence-based apps which take into account the preferences and recommendations of final users. In the present work, patients and professionals provided recommendations about app development and implementation. We hope that these results help to develop mHealth solutions that can be implemented in medical settings to better address the psychological needs of patients undergoing BS.

## Appendix 1: Guide Interview for Patients’ and Professionals’ Focus Groups

### Questions Included in the Guide for Patients’ Focus Group

What technology do you usually use in your daily tasks? Do you know how much time do you spend using your mobile phone? What are the mobile applications that you use the most? Do you have any mobile applications about mental health, nutrition, exercise, etc.? What do you know about the use of technology for mental health purposes? Do you think that psychological interventions provided through a mobile application are effective? Do you have any experience with this kind of mobile application? Do you have any experience with mobile applications for physical health purposes (i.e., calorie counting, exercise, healthy habits, etc.)? What differences do you find between face-to-face and online psychotherapy? What elements do you think that the mobile application should include to be effective? What are the main barriers and facilitators for the implementation of such technology? Would you recommend and use this mobile application to patients who have undergone bariatric surgery and are suffering emotional disorders? Do you know any strategies to promote engagement with the mobile application?

### Questions Included in the Guide for Professionals’ Focus Group

What do you know about the use of technology for mental health purposes? What is your experience with the implementation of psychological interventions through technology (i.e., telephone, video calls, internet platforms, etc.)? Do you think that psychological interventions provided through a mobile application are effective? Do you have any experience with this kind of mobile application? What are the main differences between face-to-face and online psychotherapy? What elements do you think that the mobile application should include to be effective? What are the main barriers and facilitators for the implementation of such technology? What are, in your opinion, the main benefits of using mobile applications to implement the Unified Protocol? Would you recommend to and use this mobile application with patients who have undergone bariatric surgery and are suffering emotional disorders? Do you know any strategies to promote engagement with the mobile application?

## Appendix 2: Consolidated Criteria for Reporting Qualitative Studies (COREQ): 32-Item Checklist

**Table:** 

Domain 1: Research team and reflexivity		
Personal characteristics		
Topic	Guide question/description	Page no
1. Interviewer/facilitator	Which author/s conducted the interview or focus group?	5
2. Credentials	What were the researcher’s credentials? E.g. PhD, MD	1
3. Occupation	What was their occupation at the time of the study?	1
4. Gender	Was the researcher male or female?	1
5. Experience and training	What experience or training did the researcher have?	5–6
Relationship with participants		
6. Relationship established	Was a relationship established prior to study commencement?	5
7. Participant knowledge of the interviewer	What did the participants know about the researcher? e.g. personal goals, reasons for doing the research	5
8. Interviewer characteristics	What characteristics were reported about the interviewer/facilitator? e.g. Bias, assumptions, reasons and interests in the research topic	5
Domain 2: study design		
Theoretical framework		
9. Methodological orientation and Theory	What methodological orientation was stated to underpin the study? e.g. grounded theory, discourse analysis, ethnography, phenomenology, content analysis	6
*Participants selection*		
10. Sampling	How were participants selected? e.g. convenience, consecutive, snowball	4
11. Method of approach	How were participants approached? e.g. face-to-face, telephone, mail, email	5
12. Sample size	How many participants were in the study?	4, Table 1
13. Non-participation	How many people refused to participate or dropped out? Reasons?	4
Setting		
14. Setting of data collection	Where was the data collected? e.g. home, clinic, workplace	5
15. Presence of non-participants	Was anyone else present besides the participants and researchers?	5
16. Description of sample	What are the important characteristics of the sample? e.g. demographic data, date	4, Table 1
Data collection		
17. Interview guide	Were questions, prompts, guides provided by the authors? Was it pilot tested?	6
18. Repeat interviews	Were repeat interviews carried out? If yes, how many?	5
19. Audio/visual recording	Did the research use audio or visual recording to collect the data?	5
20. Field notes	Were field notes made during and/or after the interview or focus group?	5
21. Duration	What was the duration of the interviews or focus group?	5
22. Data saturation	Was data saturation discussed?	13
23. Transcripts returned	Were transcripts returned to participants for comment and/or correction?	6
Domain 3: analysis and findings		
Data analysis		
24. Number of data coders	How many data coders coded the data?	6
25. Description of the coding tree	Did authors provide a description of the coding tree?	6
26. Derivation of themes	Were themes identified in advance or derived from the data?	6
27. Software	What software, if applicable, was used to manage the data?	6
28. Participant checking	Did participants provide feedback on the findings?	7
Reporting		
29. Quotations presented	Were participant quotations presented to illustrate the themes / findings? Was each quotation identified? e.g. participant number	Appendices 3 and 4
30. Data and findings consistent	Was there consistency between the data presented and the findings?	6–11
31. Clarity of major themes	Were major themes clearly presented in the findings?	6–11
32. Clarity of minor themes	Is there a description of diverse cases or discussion of minor themes?	6–11

## Appendix 3: Extraction of Areas, Subcategories and Categories for Patients Focus Groups

**Table:** 

Categories	Subcategories	Description	Areas	Verbatim examples
App design	Content	Areas perceived as indispensable within the app’s design	Useful information (2) Emotional regulation (5) Crisis intervention (2) Nutrition (1)	You need information all the time. Maybe at the time of the operation they tell you, but you don’t retain it” (P.2). “I should have information about the surgery and its consequences: medical and emotional” (P. 1) “After the operation you can be depressed… I was missing something” (P. 1) “Seeing that I couldn’t eat like everyone else affected me” (P. 2). “I felt sad too, but because I couldn’t get food inside me… It’s like I was missing something” (P. 4). “When I’m bored at home I think: now what do I eat… That when I have those cravings to eat something I shouldn’t, I could go into the app and it would tell me to do this. They do surgery on our stomachs, but not on our heads” (P. 1). “Everyone. Yes, the help of knowing yourself, of being able to think, to help yourself… Those modules, being able to have that help available there… It is fundamental” (P. 5) “SOS section for crisis situations. The SOS is a good thing because we will always have that problem: resorting to food” (P. 2). “It would be very good… Some people would be at it all day” (P. 5) “There could be a nutrition section” (P. 5)
	Functionality	How the components could be presented	Logs (3) Audio-visual (1) Reminders (4) Reinforcement (2) Chat-social support (4) Notes (1) Questionnaires (1) Guided (1) Personalization (3) Weight scale(1) Calendar (1) Shopping cart (1)	“Something that doesn’t require a lot of work… Another thing is if you have to record a weight” (P. 2). “There are many weight graphs that are connected to the mobile phone with apps… that measure water, weight, heart rate” (P. 5). “When you detect it (the emotion), then you would have to enter the application to…” (P. 1) “It’s not the same whether it’s a video or a reading… I read it and it’s as if they were talking to me in a language that I don’t understand” (P. 4) “Alarms going off from time to time” (P. 3). “Even an alert from time to time for a healthy recipe that you might fancy…that keeps you connected to that app” (P. 1). “Something that reminds you that the app is there” (P. 2). “Let the app tell you: you’ve been doing a year, how are you doing? ” (P. 5) “Who doesn’t like to be told: you’re doing very well, congratulations” (P. 3). “Gives you a reward that you can get easily” (P. 5) “That you can have contact via chat. You have a problem and within the app you have contact with people” (P. 5). “It would be very complicated, but a chat with a professional… A social section where you can have a chat, communicate with friends” (P. 4). “Above all, try to involve the partners more… because it is a very important foundation” (P. 5). “That the professional says in 15 days we’ll have a Webex call… So there is no need to come all the way here” (P. 1) “A blog section to write up your routines” (P. 5) “Yes (assessment), in a private section for the professional and the person” (P 0.5) “You are not going to learn everything at once, even if you put an extensive tutorial. You go inside and try it out” (P. 5) “That when I had those cravings to go and eat something I shouldn’t, I could enter the application and it would suggest something else and distract me from that thought” (P. 1). “That depending on what situation you are in, you need one function or another” (P. 2). “The possibility of putting content” (P. 4) “Always the connection with one of those weight graphs that measure percentages of fat or muscle or whatever” (P. 5) “A calendar, to plan a diet or something like that” (P. 5) “A shopping cart” (P. 5)
	Duration of monitoring	Time the app should be active during the intervention process	Before surgery (4) After surgery (5) Continuous use (3) Frequency of app use (2)	“But it would be useful even before the operation because I didn’t have any information. People who are in the pre-surgery group, post-surgery, one year after surgery, two years and more than two years” (P. 1). “The app would be very useful when a person is going to have an operation, or even if they are not going to have an operation, but they go to a psychologist because they eat too much” (P. 5). “It would be very interesting especially for people who have just had surgery” (P. 5). “The app has to be followed by everyone from the beginning and they get used to it” (P. 5) “I think that the first year is crucial” (P. 1). “Especially for people who have just had surgery, which is a time… when you are most lost” (P. 5). “For me the first year… it didn’t require a big effort. Now, however, it is a sacrifice” (P4). “It should also be designed for people who, like us, have had surgery more than three years ago” (P. 2). “People who are in the pre-surgery group, post-surgery and after one, two and more than two years” (P. 1) “The information thing no, but the SOS thing yes, because we will always have that problem, resorting to food” (P. 2). “Over the years you always fall off the wagon. The years go by and I forget those strategies” (P. 3). “It’s been 7 years now and… I will be like this all my life… Maybe if at the beginning it is more continuous, but at the end it can be more occasional” (P. 5) “At the beginning every week is fine, but later on every 15 days” (P. 2) “When time goes by, after two or three years onwards, so that it doesn’t get boring, it should be once a month” (P. 3)
	Professionals’ involvement	Professional profiles that should be involved in the operation of the app	Surgeon (1) Endocrinologist (1) Psychiatrist and psychologist (all) Dietician / nutritionist (2) Traumatologist (1)	“The surgeon before and after the surgery” (P. 3) “The endocrinologist is more dedicated to assessing analyses… they look after the physical aspect” (P. 5) “It is necessary to include a psychiatrist or a psychologist” (P. 2) (all participants agreed) “The dietician, to control and advise us” (P. 4). “The nutritionist that I had helped me a lot too” (P. 5) “It would be good if there was a traumatologist because most of us who are like this have bad knees or feet due to being overweight” (P. 5)
App implementation	Barriers	Difficulties that might exist when applying the UP in app format	App not interesting to the user (3)	“That it might bore you” (P. 1). “If you are explaining it to us we are listening, active…” (P. 2). “If you have to read everything… I am the first one whose mind… begins to wander (P. 3)
	Solutions	Solutions to barriers encountered	Audio (1) Videos (1) Gamification (1)	“In some way, if you are explaining it to us, we are listening, we are active, we participate…” (P. 2) “For me it would be much better if there is a video of the professional explaining it” (P. 4) “App that is like a game” (P. 5)
	Population profile	Population that would be recommended to use the app	General population (1)	“It’s an app that would be good for you to be healthy. For those who are diagnosed, for them, compulsory. But for others as well” (P. 5)
Opinion on technology	Usefulness	Extent of belief that the app is useful	Use for psychological treatment (5)	“If used in the right way… If we use it correctly, it can be very good. It can be very good and it can help, but only if you listen to what it tells you” (P. 1). “It would be very interesting” (P. 5). “It’s more difficult… it’s not the same as having a psychologist in person who tells you to do it” (P. 2). “It’s like the food, diets, sport and everything” (P. 3). “It’s one of the things that works very well… if you get to meditate a little bit before each meal it helps you not to eat” (P. 5)
	Face-to-face benefits	Arguments in favor of face-to-face monitoring	Commitment to therapist (4) Personalization (1) Motivation/Reinforcement (2) Credibility (1) Mobility (1)	“In the struggle for not regaining the weight lost… being accountable to someone helps a lot” (P. 1). “You are doing badly, but you have the courage to go and get weighed” (P. 3). Exemplifies a hypothetical self-verbalization: “I have done badly, but from now on…” (P. 4). “It has happened to me, I have said at times: I am not going. I did go, but I don’t know if it was just to face the music” (P. 2) “She knows about my case, but the app doesn’t” (P. 1) “The therapist always motivates you after seeing her. You come out stronger. You feel good when you go there and you have lost weight” (P. 5). “If you are face to face, that person motivates you personally, but the app is the same for everybody” (P. 2) “I think that it’s for the credibility” (P. 2) “At least this way you get out of your house” (P.5)
	Face-to-face disadvantages	Arguments against face-to-face monitoring	Shame (2) Displacement (1)	“When you know you are doing it wrong you are ashamed to go and tell him” (P. 2). “There are days when you think… he’s going to think ‘why am I going if I haven’t done it?’ ” (P. 4) “Exactly, that would be a disadvantage (the displacement) ” (P. 5)
	App advantages	Arguments in favor of app-based monitoring	Immediacy (3) Perseverance (1) Personalization (2) Distance learning (1)	“It’s easier. It’s more instantaneous” (P. 2) “If I have a doubt I go to it straight away because with you I might not have an appointment until…” (P. 3). “When you wake up, being able to have a psychologist there to tell them what is happening to you and to help you at a specific moment can be very important” (P. 5) “If you are motivated, it helps you to keep going in your daily life” (P. 1) “It would focus on the specific situation you are going through” (P. 1). “Professionals could see my graphs prior to my visit” (P. 5) “A person who can’t move around much can have direct contact at a specific moment” (P. 5)
	App disadvantages	Arguments against app-based monitoring	Veracity of data (3) Lack of personalization (1) No mobility (1)	“I can say whatever I want to the mobile phone and it won’t control me” (P. 1). “It’s more about credibility…they are selling a product” (P. 2). “It’s easier to deceive yourself with the app” (P. 5) “An app is the same for everyone. What motivates me might not motivate you and the other way round. (P. 2) “If you use the app, it’s harder to get out” (P. 5)
	Format preferences	Extent of belief that the two modalities can be complementary	Blended (all)	“It would be best after the face-to-face” (P. 1). “I think it would be perfect… You know that behind the app there is a professional that can see your responses and they will correct you” (P. 2). “Both options (online and face-to-face) combined would be the best solution” (P. 5) (P. 3, P. 4 and P. 6 agreed)
Use of technology	Type of device	Technology used daily	Mobile (all) Tablet (2) Television and computer (2)	“I use my mobile phone” (P. 4) (all participants agreed) “Tablet… Nowadays I use everything that has an Internet connection” (P. 1). “I also use the tablet” (P. 5) “I use the TV” (P. 1). “The computer is also used” (P. 5)
	Period of use	Hours spent daily on the use of technology	1 h (1) 3–4 h (2) 4–5 h (1) 10 h (1)	I spend one hour” (P. 3) “I spend 3–4 h a day” (P. 0.2). “I don’t know, maybe about 3–4 h. Maybe more” (P. 4) “Four or five hours” (P. 5) “Me 10 h. And if I can more, more” (P. 1)
	Target	Purpose for which technology is used	Information (3) Leisure time/multimedia (3) Employment (1) Social networking (3) Sport and food (2) Mental Health (2)	“No, to look for information on everything. For example, the illness of a relative” (P. 1). “looking for information on Google…” (P. 2). “I use it for various information” (P. 5) “Playing solitaire, playing games with colours, for me it’s a moment of rest” (P. 2). I play strategy games like Clash of Clan, Minecraft, sudoku…” (P. 5). “Mostly watching YouTube videos” (P. 5) “Both (work and leisure) ” (P. 5) “Instagram or Facebook” (P. 1). “Even WhatsApp chats. On Facebook there are 2–3 bariatric surgery groups” (P. 2). “I also follow those recipes that appear on Facebook and Instagram” (P. 3) “But also sports and food apps. Strava, a social network that is connected to the watch and marks a route… And it not only records what you do, but also the friends you have” (P. 1). “I downloaded some fitness and exercise apps… (WeWard) is for counting your steps, it connects you with your friends and at the end of the day it gives you points” (P. 0.5) “I download meditation podcasts on Spotiffy” (P. 2) “I had one that did meditation” (P. 5)

## Appendix 4: Extraction of Areas, Subcategories and Categories for Professionals Focus Groups

**Table:** 

Categories	Subcategories	Description	Areas	Verbatim examples
App design	Content	Materials and content that should be addressed through the app	Psychoeducation (1) Emotional regulation (6) Medical aspects (2) Useful information (3) Exercise (2)	“Teach them to differentiate between moments of real hunger and emotional hunger” (PR. 3) “So that they can work on it. So that they do not have feelings of guilt” (PR. 3). “What we see in those who have an excellent evolution: motivation, when it comes to diet, exercise and the emotional aspect” (PR. 9). “In the unit we see patients referred by the endocrinologist when there has been a problem after bariatric surgery and we do see in most cases that there is a problem that they don’t know how to manage. Many become depressed because they don’t use food to channel their feelings and they have an emptiness that if they don’t learn to regulate in another way they will relapse again when their digestive system allows it”. (PR.4). “It is true that there should be some kind of psychological follow-up after surgery. Give them guidelines to manage moments of food transgression” (PR.2). “We have little experience in this aspect because we see that psychological assistance is not covered. We have an obesity unit and hundreds of patients are operated on every year and it is incredible that they do not have psychological assistance. It is worth looking at from the point of view of a bariatric surgery unit” (PR. 5). “Post surgery there are people with many anxious and depressive symptoms, so you have to treat them in whatever way you can” (PR.1) “Thinking a bit about them and their needs, I think it should also include information on medical aspects because they are always very anxious about this subject which is very unfamiliar to them” (PR 4). “Information on how to lose weight because they think that once they have had surgery they have to be super thin” (PR. 6) “Information on how to prepare meals. Things that make their life easier” (PR. 9). “When I think of the app, I imagine that it has to include aspects on three levels: exercise, nutrition and emotional support” (PR. 4). “I know of a dietician who also included a shopping list. Products that can be bought and they make it by food recommendations” (PR. 6) “What we see in those who have an excellent evolution: motivation, when it comes to diet, exercise and the emotional aspect” (PR. 9). “When I think of the app, I imagine that it has to include aspects at three levels: exercise, nutrition and emotional support” (PR. 4)
	Functionality	Aesthetic features that should be included in the app	Logs (3) Questionnaires (1) Weight graphs (all) Audiovisual (2) Chat (1) Calendar (1)	“I think it should include daily records of how these people feel on a day-to-day basis and then the psychologist should evaluate them” (PR.8). “At times of increased risk for dietary transgressions, they can note down the moment” (PR.2). “I also think it would be interesting to record hours of sleep, stress, exercise” (PR.1) “But these types of emotional problems, of emotional regulation, do not come up unless you ask directly” (PR.10) “Visual record of weight control” (PR.4) (all participants agreed) “We can put a short podcast with guiding information because if they don’t like to read they would like that style” (PR. 9). “They can also put videos” (PR.9). “Let it be like WhatsApp” (PR. 6) “That they can talk to each other, those who are included in this application” (PR. 6) “Remind them of the time since surgery. ‘It’s been a year since the surgery’ or ‘it was two years ago’. I would say ¡Warning! you are on the red line’, because that is when they start to get overconfident. Then remind them where they are post-surgery” (PR. 3)
	Duration	Importance of timing and duration of intervention	Time of intervention (3) Duration of monitoring (2)	“It makes sense post and pre (…) Post there are people who have many anxious and depressive symptoms, so you have to treat them in whatever way you can” (PR.1). “In post, unlike in pre, it is the excessive worry about regaining that weight” (PR. 1). “Post-surgery, as opposed to pre, it is the excessive concern they have about regaining that weight” (PR. 3) “Two years from the point of view of nutritional intervention but the monitoring is for five years” (PR.5). “After two years, it is when there is again an increase in weight (…) and it is true that it should be done in some way” (PR. 2)
App implementation	Barriers	Difficulties in the implementation of the app	Overload (2) Motivation (1) Lack of human contact (1)	“This is already getting a bit out of hand because diet, emotions, exercise are too many things to assess manually” (PR. 1). “It is also not a huge workload because if you are going to require the patient to record everything, they are still going to hate the application. The danger of this is that if they see this information and are not interested, they may close the application and not continue” (PR. 5) “Motivation, as in everything. Sometimes they don’t do very simple things. So even more so they have to be aware that they have these emotional problems. The key to the application is that they want to use it” (PR.1) “I also think that if they don’t have someone at the foundation as a reference, it can be more difficult for them to connect afterwards. If there is no psychologist behind the application, for example, who can provide them with a certain level of security, it is more difficult for them to engage” (PR. 4)
	Solutions	Strategies to improve the application response rate	Initial professionals’ contact (2) Explain benefits (2) Testimonials (2) Notifications (1) Gamification-Reinforcement (2) Personalization (3) Professionals (1)	“It is important to have a face-to-face meeting first and the importance of managing emotions first because that may be what engages them with the app. If you don’t work on managing emotions first, they may not see it as useful. That is why I think it is important to manage the previous phase” (PR. 5). “But if they are referred to a unit where psychologists could make that assessment and intervention and have the accompanying application. It is explained to them that the intervention includes follow-up and the use of the app” (PR.4) “Within the psychoeducation workshops we do, a workshop could be dedicated to taking advantage of the emotional part and all the benefits they can get from the use of this app” (PR.5). “We should introduce it in some way as a package and tell them: later on after surgery, as there are emotional problems that have to do with food intake, there will be an app” (PR. 10) “Seeing other people. I mean, there are patients who have already had surgery, have been followed up and so on. Seeing someone for whom it has worked helps a lot” (PR. 2). “That there is a patient who is an expert” (PR. 7) “That it send you a message and you don’t have to remember to log into the app” (PR. 6) “And like the clocks that when you have achieved ten thousand steps it is like a party” (PR. 2). “Sure, you have to give them reinforcements” (PR. 5) “Of course, I also think that they can have medical, dietary, psychological aspects and that they can have access to what they need at any given moment, although there may be a general basis with the register. But that this basic information in the app can be accessed if each person needs it” (PR. 4). “Written information that may be in a part of the application that is not compulsory. That it is included in the application in a section as if it were a library where you can consult something at some point” (PR. 2). “Creating your own avatar seems to work on a motivational level. Many applications get people hooked because of that part, because people identify with their avatar” (PR. 4) “And the part that is supported by professionals, I think it is important. That there is a multidisciplinary team of professionals behind the app. It will always give more confidence” (PR.4)
	Intention to use	Intention of professionals to recommend this technology to their patients	Recommendation (1)	“We would have to see how it works first” (PR. 2)
Opinion on technology	Usefulness	Extent of belief that the app is useful	Use of apps (2)	“I do know of applications that are good and I use them with patients” (PR.7). “Yes, if we see that an individual patient can benefit from a particular application, we might give it to them as a recommendation” (PR.3)
	Face-to-face disadvantages	Disadvantages of face-to-face emotional assessments	Biases (1) Time (1)	“So sometimes the information they give you is very biased and you have to be there checking and checking and you don’t trust everything they tell you” (PR. 2) “Of course, because you get a first visit and you don’t know them and in half an hour you have to decide whether this person is fit or not to be operated on. So it often makes little sense to what extent I know if this patient can be operated on. We all know that any assessment requires several sessions and that is physically impossible” (PR. 2)
	App advantages	Arguments in favor of app-based monitoring	Accessibility (2) Self-monitoring (1) Information (1)	“A more continuous monitoring, that they have it on a more conscious level” (PR.4) “Easier access to information” (PR.7) “I think with the help of the motivational part as well, so that they can keep their own monitoring” (PR.4). “I think that in this sense the app for monitoring emotions” (PR.5) “I think the advantage of the app would be that all the medical part would be in writing: the most frequent, the least frequent” (PR. 4)
	Format preference	Extent of belief that the two modalities can be complementary	Face-to-face (2) Blended (4)	“Right now I think the need is to have someone who can help with these symptoms, like a psychologist or psychiatrist, to help regulate them. And to do this through a mobile app, I personally don’t see it” (PR.1). “We need that visual positive reinforcement. Yesterday I had a patient who had bariatric surgery but she didn’t believe it and I told her how much weight she has lost. That’s why they do need that personal contact” (PR.6) “Maybe it would be a second phase. In the first phase they would need a more personal contact and then probably something complementary would be possible” (PR.5). “The idea is a bit of a blended system, which is a combination. Given that we can’t see them as often as they would need, if we can see them once a month they would have all those guidelines that can help them, dietary aspects, a thermometer of emotional regulation” (PR.4). “I think it is a way of having that contact that we can’t maintain due to lack of staff and it is a way for patients to have that contact until the visit” (PR.2). “What we do here is mostly individualised and combined with group sessions” (PR.3)

## Data Availability

Data supporting the findings of this study are available within the article and its supplementary files.
